# Prescriptions for the Control of a Clonal Invasive Species Using Demographic Models

**DOI:** 10.3390/plants11050689

**Published:** 2022-03-03

**Authors:** Gabriel Arroyo-Cosultchi, Jordan Golubov, Jonathan V. Solórzano, Maria C. Mandujano

**Affiliations:** 1 Programa de Botánica, Colegio de Posgraduados, Km. 36.5 Carr. México-Texcoco, Montecillo, Texcoco 56230, Mexico; gcolultchi@gmail.com; 2 Departamento de Ecología y Recursos Naturales, Facultad de Ciencias, Universidad Nacional Autónoma de México, Ciudad Universitaria, Mexico City 04510, Mexico; 3 Departamento El Hombre y Su Ambiente, División de Ciencias Biológicas y de la Salud, Universidad Autónoma Metropolitana–Xochimilco, Calz. Del Hueso. 1100, Col. Villa Quietud, Mexico City 04960, Mexico; 4 Centro del Cambio Global y la Sustentabilidad en el Sureste A.C., Calle Centenario del Instituto Juárez, S/N, Col. Reforma, Villahermosa 86080, Mexico; jgolubov@gmail.com; 5 Instituto de Ecología, Universidad Nacional Autónoma de México, Circuito Exterior s/n, Ciudad Universitaria, Mexico City 04510, Mexico

**Keywords:** herbicide treatments, integral population models, *Kalanchoe*, plantlet survival

## Abstract

Until recently, little research has focused on determination of the population dynamics of invasive species and evaluating their genetic variation. Consequently, not much is known of what drives clonal invasive species and their demography. Here, we describe the population dynamics of *Kalanchoe delagoensis* (Crassulaceae), considered invasive to several countries. We quantified the demography of a population in central Mexico using integral projection models (IPM) in a population that reproduced asexually exclusively through plantlets. The effect of clonal recruitment on population growth rate (λ) was evaluated by changing plantlet survival and simulating management scenarios that used previous data of watering and seven experimental herbicide treatments. The finite rate of population increase indicated that this *Kalanchoe delagoensis* population is growing (above one) and with water availability, growth rates will only accelerate. The IPM showed that plantlet survival and recruitment were the most critical steps in the cycle for the population, and simulations of different management scenarios showed that reducing plantlet survival significantly decreased λ only in two out of the seven herbicides used.

## 1. Introduction

Interest in invasive alien species (IAS) has grown in recent years, as the impacts are widely recognized [1], and global costs are being quantified [2,3,4]. Critical stages of invasion involve the establishment and spread into new habitats (categories C, D, and E in [5]), which can depend on a suite of factors including habitat suitability [6], components of propagule pressure (frequency and size of introductions [7,8]), presence/absence of pollinators, dispersers, or herbivores [9], and phenotypic plasticity [10,11]. The reproductive mechanisms behind the spread can vary widely, however, even though clonality seems to be quite common among invasive alien species [12], the role played by clonality during spread is context-dependent [13,14]. Of the 31 papers we reviewed that deal with population dynamics of invasive alien species, six had some form of vegetative reproduction [15,16]. Even though no attempt has been made to evaluate the overall demographic importance of clonality among invasive species, it is widely recognized as an essential trait in risk assessments [17,18,19] and invasion success [14] and is a critical component of population growth [20].

Only recently have demographic models been used to assess components of invasion and to guide management of invasive species [15,21,22,23,24,25], which can even include prioritization based upon cost [26]. Population dynamics of IAS commonly have high population growth rates [15], and transient dynamics seem to play a part in invasion success [16,27,28]. Invasive species have also been shown to have demographic plasticity [29,30] and buffer environmental variation [21,31], which enables them to exploit both ends of the phenotypic plasticity spectrum [master of one, jack of all [10]] and even combinations of these [32]. Targeting certain life stages (or combinations thereof) can provide insight towards identifying critical pathways [22,25,33,34] that would lead to optimal management strategies [15,27], as well as diminish the cost and overall impact of IAS [35].

Integral projection models (IPMs) have been used recently and widely for demographic analyses (e.g., [36,37]), including assessments of population viability for endangered plants (e.g., [38]) or invasive plants (e.g., [24,25,31]). Demographic modeling can then provide insight into short-term possible directions for management, and they benefit from being robust to small data sets compared to matrix population models [39] and are able to incorporate other explanatory drivers as covariates [31,38,40].

The genus *Kalanchoe* is native to the dry regions of Madagascar and Eastern Africa, and several species are now found in several countries, where it has been introduced and spread either accidentally or through the horticultural trade [41,42]. Populations of *K. delagoensis* in Mexico share a single genetic makeup suggesting a single introduction event with subsequent spread [43]. Even though herbicidal control has been tried, as have controlled burns, management has been difficult due to the high production of clonal plantlets from the margin of leaves. This apomictic trait is the main driver of population dynamics of its congener *K. daigremontiana* in Venezuela [44].

The aims of this study were to (1) assess the population dynamics of *K. delagoensis*, and (2) determine the management effectiveness of targeting the plantlet life stage which has been previously shown to be susceptible to some herbicides.

## 2. Results

### Vital Rates

**Figure 1:**
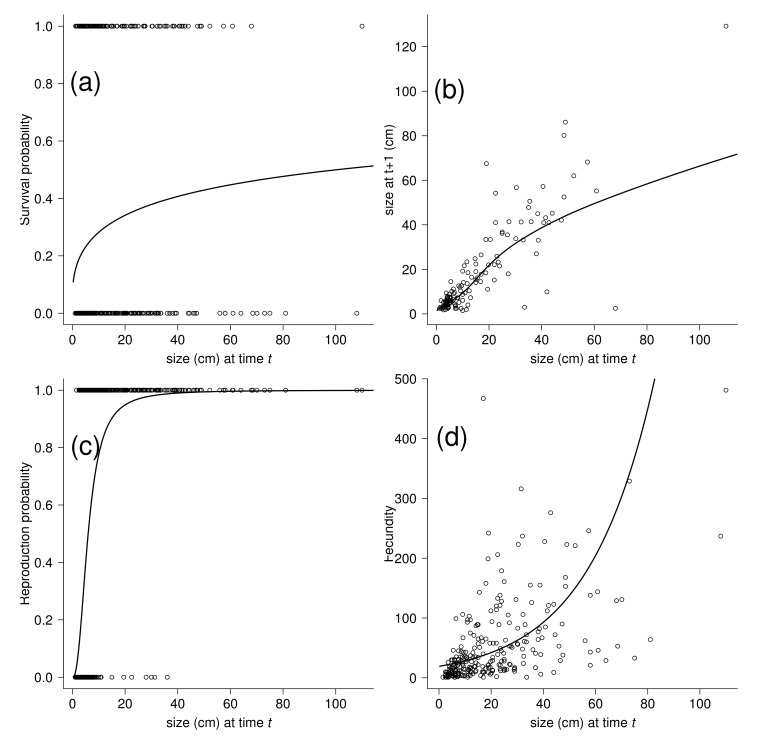
Fitting of the survival, growth, reproduction probability, and fecundity of the *K. delagoensis* 2011 data. (**a**). The survival (s) data are plotted (0, death; 1, survival) as a function of individual size *x* (plant height in cm), along with a logistic regression fitted to the data. The fitted curve is log(s/(1 − s) = −1.904 + log(0.206)*x* (*p* < 0.05). (**b**) The data on year-to-year changes in size, along the regression fit for mean size at year *t* + 1 as a function of size in year *t*. The fitted line has μ = 0.327 + log(0.885)*x* (*p* < 0.05) and $\sigma^{2}$ = −1.273 + log(0.269)*x* (*p* < 0.05). (**c**) The reproductive (r) probability is plotted (0, nonreproductive; 1, reproductive) as a function of individual size *x* (plant height in cm), along with a logistic regression fitted to the data. The fitted curve is log(r/(1 − r) = −4.316 + log(2.414)*x* (*p* < 0.05). (**d**) The fecundity as a function of individual size, along with the regression for the mean number of plantlets. The fitted line is μ = 2.955 + log(0.039)*x* (*p* < 0.05). The *y*-axis scales are different among the panels.

A total of 497 individuals were followed during the study period. Models adjusted for survival, fecundity, and growth to generate the kernel used a log transformation of plant size to improve fit. Of the models fitted to the data (survival with five models, growth with six models, reproductive probability with six models, and fecundity with five models), those with the lowest value of AICc were used (Figure 1; Table S1 Supplementary File). The production of plantlets was found even for small individuals that started to produce plantlets within the year and with a plant size as small as 1.5 cm. The population of *K. delagoensis* studied showed no seeds; therefore, the driver of population growth was entirely through the proliferous production of plantlets. During the period (2010–2011), the survival probability of *K. delagoensis* and growth (regression with slope > 1; Figure 1a,b) increased with stem size, and probability of reproduction and fecundity also increased with stem size (Figure 1c,d). In our study site and period, the population growth rate (λ) of *K. delagoensis* with plantlet survival set for the control treatment (no water and full sunlight) was > 1 (Table 1), which would suggest the growth rate under severe natural conditions. Fecundity (Fmatrix) contributed 60% and 52% to the population growth rate (λ) for 100% water and other herbicide treatments, respectively. The high values of λ (3.51 and 2.08) of these models suggest the importance of vegetative reproduction in the dynamics of the population and the potential for invasion. Survival-growth (Pmatrix) contributed 100% and 78% towards λ for G/2-4D and 2-4D treatments, respectively, basically due to the high plantlet mortality caused by the herbicide treatments. Herbicide treatments (G/2-4D and 2-4D) contributed to the reduction in λ and point towards an effective control of *K. delagoensis* populations that rely on clonal reproduction. Although not considered here, the effects of plantlet mortality could be offset by high water availability, which suggests herbicide treatments should be avoided during rainy seasons to maximize plantlet mortality.

## 3. Discussion

Demographic variation at an individual level has only recently been taken into account to determine how individual traits influence population growth [22,45]. Clonality is one of those components that for invasive species seems to be a known and important driver of invasion [14,34,46,47,48]. However, little attention has been given to the demography of clonal invasive species, and the role played by the clonal component, even though it may be the only means of population growth for some extremely clonal species. This lack of information could be partly due to the cost and difficulty of differentiating genotypes in natural populations of clonal species or to the often overriding importance and haste of direct management options and/or eradication. Nevertheless, among invasive alien species, some examples exemplify the role played by clonality [43,47,49]. Fortunately, sexual reproduction has not been found in the studied population of *K. delagoensis*, which the genetic data also suggest [43]. Adding seeds would increase genetic diversity and quite possibly generate even broader responses to environmental variation and increased invasive capacities [49]. Sexual reproduction would also very likely create seed banks such as those found for *K. daigremontiana* [50] complicating management further and increasing management costs basically because of the longer control periods needed to reduce the seed bank. Clonality may not have the potential for dormant stages nor the benefit of genetic variability, but clonal offspring can have higher survival rates over long periods and be a strategy to occupy suitable habitats in short time periods.

Almost all of the invasive species with some form of clonality have population growth rates above equilibrium [15,51,52,53,54]. This observation is consistent with the spread of invasive species [5] and is one of the reasons for highlighting the importance of clonality in risk assessments. Clonality coupled with other traits such as self-compatibility [55], resprouting [22], and no need for pollinators or dispersers makes these species more likely to invade successfully than others. There are clear benefits of clonality including the spread of an already successful genotype for a determined habitat. If the traits of clonal offspring have phenotypic plasticity, the species can exhibit a “Jack and Master” strategy [10] allowing the possibility of increasing fitness in favorable environments while maintaining fitness in stressful environments. This would also be compounded by complex transient dynamics [16,28] and variation in vital rates [31]. Even small increases in favorable abiotic conditions have the ability to add to plantlet survival and, therefore, increase the invasive potential significantly.

### Management Scenarios

A successful management option suggested by [15] was the reduction in fecundity for short-lived invasive species, but seemingly that cannot be applied consistently across species [56]. Reducing one of two demographic processes (fecundity or stasis) was shown to be sufficient to control growing populations [15]. For the sister species *K. daigremontiana* and *K. pinnata*, clonality, as well as sexual reproduction, contributed significantly to population growth [34,57]; however, the genetic identity of the individuals was not evaluated. The lack of a sexual component of fecundity in *K. delagoensis* at the site makes control easier and also highlights the danger of triggering higher growth rates if genetically dissimilar individuals are introduced. Ramula et al. [15] also found that reducing one demographic process (namely growth or reproduction) under simulated conditions was adequate to control short-lived invasive species, while reduction in at least two was needed to control long-lived perennials. Knight et al. [56] simulated reduction in the sexual component of perennial invasive species and found that reducing this demographic process was not enough to curb population growth. As the plantlets of *K. delagoensis* contribute to fecundity, a management scenario would necessarily involve lowering the recruitment of plantlets by using one of two herbicides that had important effects on plantlet survival. Unfortunately, not all herbicides had the same effectiveness, and of the seven that were tested, only two induced high enough mortality in plantlets to generate the falls in λ needed for adequate control. The rest of the herbicides had plantlet survival rates that did not affect λ sufficiently to be considered an effective control. Lowering recruitment of plantlets, however, poses a problem because plantlets are produced by very young individuals and are generated year-round, such that management must be carried out continuously and would entail long-term control. The timing of herbicide applications should also consider that mortality can be offset in the seasons with higher water availability.

The populations of *K. delagoensis* in Mexico pose an interesting system in which plantlets are the drivers of population growth. This also means that management can be feasible if plantlet survival is severely limited in this population. Little is known of the demographic behavior in other populations, which could potentially behave very differently and increase the invasion potential of this and the sister species. Currently, only *Kalanchoe pinnata* and *K. delagoensis* are listed as invasive species in Mexico [58], but the sister species *K. daigremontiana* and what is known as Houghton’s hybrid (*K. diagremontiana* × *K. delagoensis*) already have large populations with wide distributions and also pose a significant risk as they have very similar demographic behavior.

## 4. Materials and Methods

### 4.1. Species

*Kalanchoe delagoensis* Ecklon & Zeyher [59] is a succulent able to reproduce sexually as well as clonally through pseudobulbils (hence the name “mother of millions”) that arise from the margin of their leaves [60]. Clonal plantlets have accelerated growth and high survival, and are a common sight in invaded areas [61]. *K. delagoensis* is considered to be an aggressive invader in Australia and the USA [41]. It is included in the exotic species lists in Mexico, and risk assessments suggest a significant risk to native vegetation and soils (Guerrero-Eloisa, unpub. data).

### 4.2. Population Dynamics

For two years (2010–2011), a population of *K. delagoensis* (20.687061° N, −99.805255° W) was followed. The site has 15.9 °C average annual temperature and 488 mm annual rainfall (data from 60 years obtained from the National Meteorological Service stations at the site). Vegetation at the site consisted of Chihuahuan xerophytic scrub. During April 2010, seven 1 × 1 m plots were set, and all individuals of *K. delagoensis* within the plots were tagged and measured (plant width (cm) and height (cm)). Tagged individuals were followed for a year.

### 4.3. Population Growth Rate

Demographic data on *K. delagoensis* were obtained as plantlets and adults. Data were widely examined for errors and outliers. All data were used to construct informative models for vital rates (individual survival, growth, and fecundity) based on plant size (height, cm) over a period of one year. We performed model selection using Akaike Information Criterion corrected for small sample sizes (AICc; [62]) to choose the most plausible models among a range of different models (Table S1 Supplementary file). The vital rate models support the basis for building integral projection models [36,37,63] to model the life cycle of *K. delagoensis* ([64]). We used an integrative measure (height, cm) as the size variable and analyzed the models, assessing the effect of size on survival and growth over the time period (one year), probability of reproduction, and number of plantlets produced per individual using generalized linear (GLM) and generalized additive models for location scale and shape (GAMLSS).

The IPM incorporates models for individual survival, changes in size, and fecundity. The models for individual survival used a logit link function GLM and binomial distribution using the lme4 package [65]. We modeled plant growth for year *t* + 1 (2011) in relation to plant size from year *t* (2010) with nonparametric normal models using the gamlSS package [66]. We estimated the size-dependent fecundity of reproductive individuals as the product of the size-specific probability of successfully producing plantlets (estimated with a logit link GLM and binomial errors using the lme4 package [65]), and the size specific number of plantlets produced (with nonparametric normal models using the gamlss package [66]). This gives an estimate of the number of plantlets produced by plants of different sizes as a means of reproduction. For the description of all the models, see the Supplementary information. The mesh size was set to 500 bins. We numerically integrated the demographic kernel using the midpoint rule to generate the IPM [36]. The dominant eigenvalue of the square matrix coincides with population growth rate (λ). Population growth rates = 1 show population stability, <1 indicate a population expected to decline, and rates > 1 indicate a growing population over the long term. Confidence intervals (95%) for λ were obtained by bootstrapping, such that individuals were resampled to generate 1000 parameters for each element in the kernel [67].

Possible management scenarios were simulated by changing the survival rates of plantlets. Plantlet survival under different treatments with watering levels and the use of herbicides was used from data taken from [68]. The baseline plantlet survival was the survival of plantlets with no watering and under full sunlight, similar to what would be expected under natural conditions. To assess the contributions of changes in vital rates to λ following management scenarios, we modified the probability of plantlet survival to what was found in the herbicide treatment experiments. We modeled 3 water treatments (25, 50, and 100% water field capacity) to determine the effect of water on population growth rates and seven herbicide treatments, five of which were grouped into those with more than 33% plantlet survival and those that had the highest mortality (G/2-4 Glyphosate + 2-4D amine mixture and 2-4D, see concentration details in [68]). Population growth rates and error intervals (through boostrapping) were calculated for each treatment. We calculated the population growth rate and the vital rate elasticity and sensitivity for the additive sub-matrices of survival-growth (P) and fecundity (F) [69] based on each IPM representing different management scenarios (elasticity and sensitivity ::popbio; [70]). 

## Figures and Tables

**Table 1:** Probability of survival after water and herbicide treatments were applied and the resulting population growth rate with their confidence intervals (95%) and the elasticity sub-kernel contributions.

Treatment ^1^	Proportion	λ	Pmatrix	Fmatrix
	of Plantlet Survival	(Confidence Interval)		
Control ^2^	0.12	1.28 (1.094–1.640)	0.556	0.444
25% Water	0.14	1.37 (1.228–1.836)	0.545	0.455
50% Water	0.25	1.81 (1.531–2.869)	0.502	0.498
100% Water	0.84	3.51 (2.234–5.142)	0.399	0.601
G/2-4D	0	0.29 (0.282–0.415)	1.000	0.000
2-4D	0.01	0.55 (0.245–0.717)	0.776	0.224
Other herbicides	>0.33	2.08 (1.860–2.410)	0.480	0.520

^1^ Survival probabilities from experiments performed in Guerra-García et al. 2018. ^2^ No water full sunlight.

## Data Availability

The data presented in this study are available upon request from the first author.

## Supplementary Materials

The following supporting information can be downloaded at: https://www.mdpi.com/article/10.3390/plants11050689/s1, Table S1: Selection of the most plausible GLM, GAMM, and GAMLSS models, predicting survival, growth, probability of reproduction, and fecundity for *Kalanchoe delagoensis*. The model selected was denoted in bold. *df* = degree freedom; AICc = Akaike Information Criterion corrected for small sample sizes; s = smooth terms; cs = cubic spline; sigma.fo = sigma.formula.
