# Supplementary material for: Prescriptions for the Control of a Clonal Invasive Species Using Demographic Models
Source: Plants (Basel). 2022 Mar 3;11(5):689. doi: 10.3390/plants11050689 (PMC8912375; doi:10.3390/plants11050689)
Supplement: Supplementary file 1 [file plants-11-00689-s001.zip › plants-1534582-supplementary.pdf]

Table S1. Selection of the most plausible GLM, GAMM, and GAMLSS models, predicting survival, growth, probability of reproduction, and fecundity for *Kalanchoe delagoensis*. The model selected was denoted in bold. *df*= degree freedom; AICc = Akaike Information Criterion corrected for small sample sizes; s = smooth terms; cs= cubic spline; sigma.fo= sigma.formula.

Model	*df*	AICc	Models
**Survival** (binomial distribution)			
**Survival~log(height + height ^{2})**	**494**	**566.70**	**GLM**
Survival ~ height	494	574.40	GLM
Survival ~ height ^{2}	494	574.40	GLM
Survival ~s(log(height)	494	568.26	GAMM
Survival ~s(log(height + height ^{2}))	494	568.65	GLMM
**Growth (**smoothing, cubic splines, normal distribution**)**			
**log(height(t+1))~cs(log(height)),sigma.fo=~cs(log(height))**	**124**	**227.81**	**GAMLSS**
log(height(t+1))~log(height),sigma.fo=~cs(log(height))	127	232.18	GAMLSS
log(height(t+1))~cs(log(height)),sigma.fo=~log(height)	127	232.18	GAMLSS
log(height(t+1))~log(height),sigma.fo=~log(height)	130	238.41	GAMLSS
log(height(t+1))~cs(log(height))	128	250.76	GAMLSS
log(height(t+1))~log(height)	131	254.24	GAMLSS
**Reproductive Probability** (binomial distribution)			
**Reproduction(t+1)~log(height)**	**494**	**392.50**	**GLM**
Reproduction(t+1)~log(height+height^2)	494	393.40	GLM
Reproduction(t+1)~s(log(height))	493	394.55	GAMM
Reproduction(t+1)~s(log(height+height ^{2}))	493	395.40	GAMM
Reproduction(t+1)~s(height)	493	405.10	GAMM
Reproduction(t+1)~ height	493	433.40	GLM
**Fecundity** (number of plantlets produced), (poisson (*PO*) or negative binomial distribution (*NBI*))			
**plantlets ~ height (*NBI*)**	**269**	**2601.31**	**GAMLSS**
plantlets ~cs(log(height)) (*PO*)	267	11924.00	GAMLSS
plantlets ~cs(height) (*PO*)	267	12099.50	GAMLSS
plantlets ~log(height) (*PO*)	270	12292.10	GAMLSS
plantlets ~ height (PO)	270	13834.00	GAMLSS
